# Eyelid Molluscum Contagiosum: A Sign of Advanced HIV Infection

**DOI:** 10.7759/cureus.53783

**Published:** 2024-02-07

**Authors:** Valeria Ortega, Roger Lovell, Diego Vanegas Acosta

**Affiliations:** 1 Medicine, Baylor College of Medicine, Houston, USA; 2 Infectious Diseases, Piedmont Athens Regional Medical Center, Athens, USA; 3 Medicine, College of Medicine, University of Florida Shands Hospital, Gainesville, USA

**Keywords:** aids-defining illness, hiv-associated neurocognitive disorders, delayed diagnosis, hiv and aids, molluscum contagiosum

## Abstract

HIV infection can present with dermatologic pathologies, and molluscum contagiosum in the eyelid is a specific sign often related to advanced HIV. Herein, we present the case of an adult patient with eyelid molluscum contagiosum that had been present for the last five years. Despite previous healthcare evaluation, the patient was not tested for HIV infection until presenting to our healthcare facility. The patient was diagnosed with AIDS. Our case highlights the importance of recognizing indirect signs of immunosuppression and the need to promptly order an HIV test. Additionally, we want to emphasize the importance of raising awareness about routine HIV testing, aiming to reduce the social stigma surrounding this test.

## Introduction

Current public health guidelines recommend that persons between 13 and 64 years old have at least one HIV test. Others should be tested annually based on certain risk factors [1]. HIV testing is recommended because early diagnosis will allow for prompt institution of antiretroviral therapy (ART) which is associated with decreased morbidity and mortality from HIV infection and its associated complications and with decreased transmission to sexual partners. Achieving and maintaining a plasma HIV RNA below 200 copies/mL with ART can prevent sexual transmission to partners [2]. Despite these areas of progress in the management of HIV infection, it is important to recognize that it remains a significant global health issue with varying prevalence across different regions of the world associated with varying levels of access to healthcare. At the end of 2022, WHO data revealed that there were approximately 39 million individuals worldwide who had been diagnosed with HIV infection [3]. Failure to diagnose HIV infection prior to the onset of significant immunosuppression is less common but is still an ongoing challenge. The late stages of HIV infection not only result in severe debilitation and comorbidities but also carry a higher risk of transmission related to elevated viral loads. Also, the differential diagnosis in patients presenting to the hospital in the late stages of HIV infection becomes challenging because of significant immunosuppression which can alter the patient presentation and result in a higher utilization of healthcare resources. Physicians must be vigilant in recognizing indirect signs that may indicate an underlying immunodeficiency such as an uncommon presentation of an ordinarily common infection.

## Case presentation

**Figure 1:**
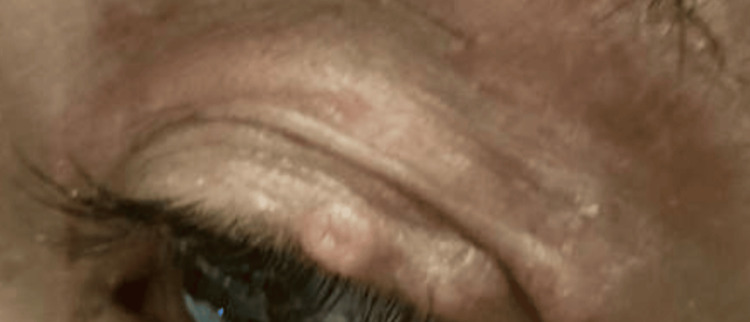
A 3-mm skin-to slightly pink-colored papule with central umbilication on the left upper eyelid suggestive of molluscum contagiosum.

A 49-year-old female presented to the Emergency Department for worsening dizziness. She had increasing difficulty in ambulation, weight loss, cough, and memory loss over the last 6 months. She reported asymptomatic face lesions, which have been present for the past 5 years. Previously evaluated by an outside dermatologist, the patient did not recall the recommended topical treatment. She denied chronic medical conditions. The patient reported unprotected sexual intercourse and no history of drug abuse. On examination, vital signs included blood pressure 99/62 mmHg, temperature 36°C, and pulse 110 bpm. Weight was 50 kg, BMI 20 kg/m^2^. A 3-mm skin to slightly pink-colored papule with central umbilication, consistent with molluscum contagiosum (MC), is present on the left upper eyelid margin, along with several similar lesions on the face that have been present for the past 5 years (Figure 1).

No other MC lesions were found on other parts of the body. She denied any problems with her vision. She had erythema and flaky scales symmetrically distributed on her face, involving mostly the malar region, eyebrows, center of her forehead, and ears. It was also extending to the scalp, consistent with seborrheic dermatitis. Oral examination showed white patches on the tongue, inner cheeks, and gums. No palpable adenopathy or organomegaly were present. There was muscular atrophy. Neurologic examination demonstrated saccadic eye movements, mild dysarthria, poor memory, and decreased attention. Motor strength was 5/5 throughout except 4+/5 in the left hip flexor. Reflexes were 3+ throughout with downgoing Babinski responses bilaterally. A tremor of high frequency low amplitude greater in right than the left hand with component of dysmetria vs physiologic tremor. The patient was aware of self, year, and situation, but not location.

The patient had a positive fourth-generation HIV antibody/antigen test with HIV-1 genotype and no mutations. Patient stated that an HIV test was not previously performed. CD4+ lymphocyte count was 4.9% (<20 cells/µL) and the HIV viral load was >1.25 million copies.

Laboratories showed anemia (hemoglobin 7.8 g/L), leucopenia (white blood cells of 2,400 cells/μL with an absolute lymphocyte count of 200 cells/μL), and normal platelet count (249,000/μL). Low albumin of 3.0 g/dL, normal total protein of 7.2 g/dL, normal levels of lactate dehydrogenase of 216 U/L, and serum glucose of 88 mg/dL.

**Figure 2:**
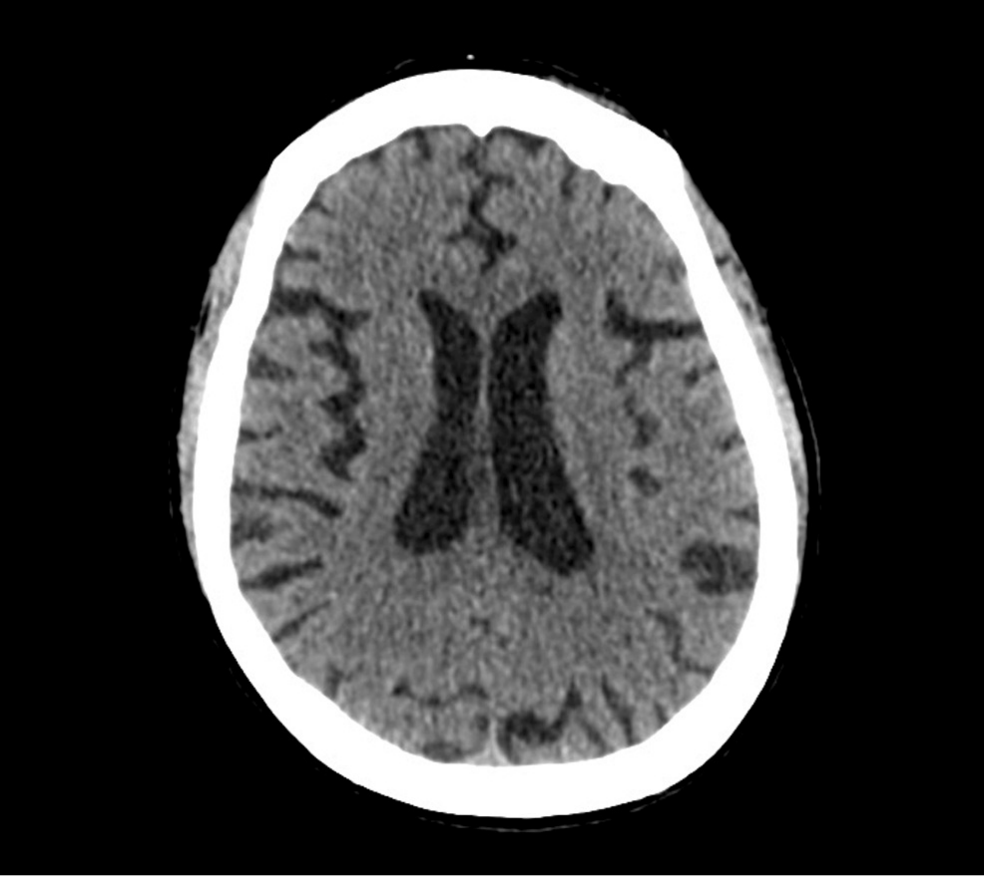
Computed tomography of the brain showing marked diffuse symmetric cerebral atrophy.

Non-contrast computed tomography (CT) of the brain revealed diffuse symmetric cerebral atrophy without significant white matter disease, focal parenchymal lesion, intracranial hemorrhage, or hydrocephalus (Figure 2).

**Figure 3:**
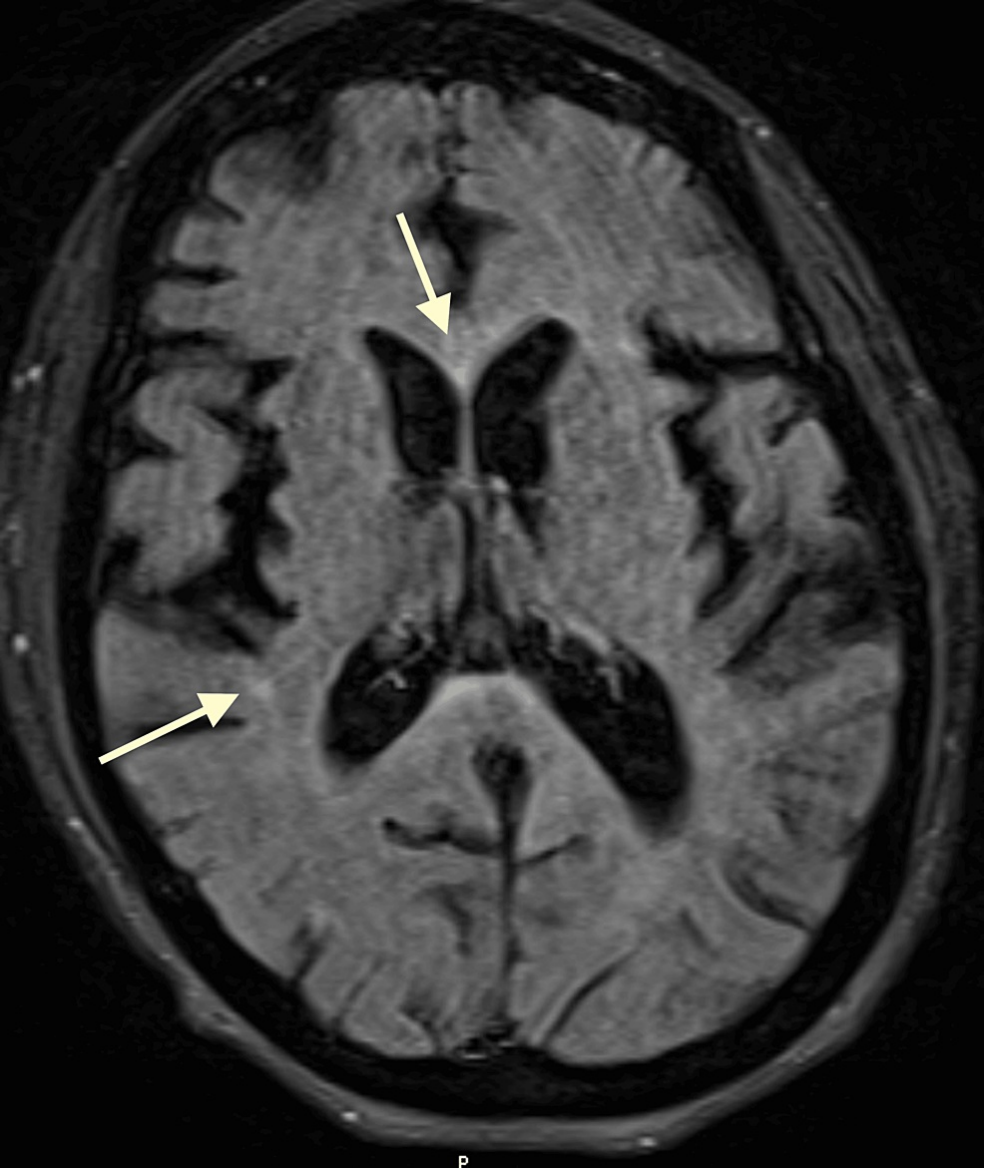
T2/FLAIR sequence magnetic resonance imaging showing asymmetric mild hyperintensities in the supratentorial white matter. Predominantly the periventricular (yellow arrow) and subcortical areas (white arrow). FLAIR: fluid attenuated inversion recovery

For further characterization, magnetic resonance (MR) was recommended. T2/fluid-attenuated inversion recovery (FLAIR) sequence showed asymmetric mild hyperintensities throughout the supratentorial white matter, predominantly in a periventricular and subcortical distribution (Figure 3).

Progressive multifocal leukoencephalopathy (PML) and HIV-associated neurocognitive disorder (HAND) were considered as potential diagnoses. While both conditions share white matter disease as a hallmark, their primary imaging distinction lies in the distribution pattern. PML typically presents with asymmetric and focal cerebral white matter changes, whereas HAND exhibits diffuse and symmetric alterations. Notably, cerebral atrophy, uncommon in PML, is associated with HAND [4,5].

Diagnostic testing on CSF was performed (Tables 1-2).

The observed cerebral atrophy, coupled with the negative JC virus PCR results, makes HAND the more likely diagnosis.

**Table 1:** CSF analysis CSF: cerebrospinal fluid

Component	Result
Appearance	Clear
White cell count	0 cells
Red cell count	3 cells
Protein (mg/dL)	56
Glucose (mg/dL)	48
Parvovirus B19 DNA PCR	Not detected

**Table 2:** Most relevant CSF infections CSF: cerebrospinal fluid; VDRL: Venereal Disease Research Laboratory; PCR: polymerase chain reaction

Test	Result
JC virus PCR	Not detected
Herpes simplex virus 1/2 DNA PCR	Not detected
VDRL	Non-reactive
Parvovirus B19 DNA PCR	Not detected
Acid-fast bacilli culture	No growth
Cryptococcal antigen	Negative
Routine bacterial culture	No growth

Other diagnostic testing included PCR for CMV (Cytomegalovirus) and Epstein-Barr virus (EBV) in the patient’s blood. Her PCR for CMV was elevated in blood (1033 IU/mL). Ophthalmology was consulted to evaluate for the presence of CMV retinitis. No CMV retinitis was present; however, a diagnosis of HIV retinopathy versus noninfectious microvasculopathy was reported. Since there was no CMV retinitis, the initiation of ART is typically adequate in managing CMV viremia [6]. EBV PCR revealed < 390 copies/mL.

**Figure 4:**
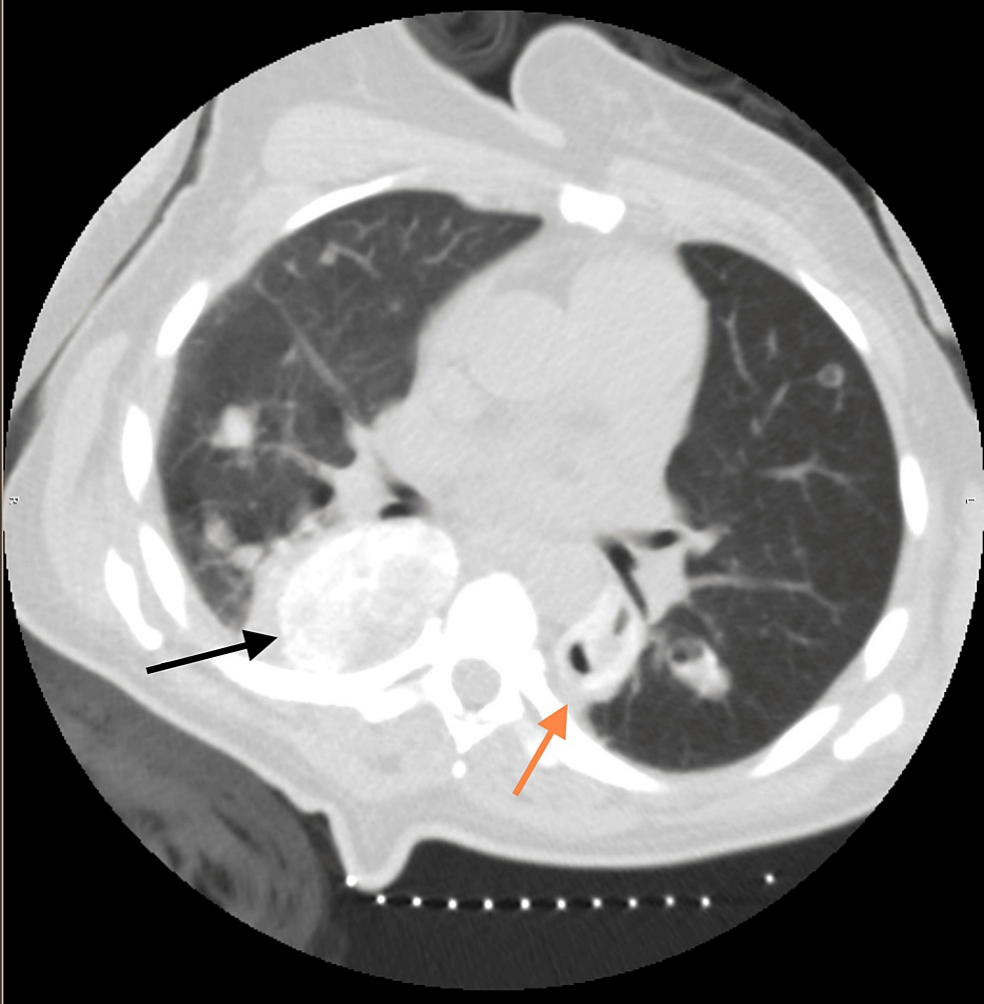
Computed tomography of chest showing multiple nodules and masses in the lower lobes bilaterally. On the right, the largest lesion measures 6.1×4.6 cm (black arrow) and on the left 2.4×1.7 cm (orange arrow). These masses appear hypervascular and with cavitation.

Blood enzyme immunoassay for syphilis was negative. Nucleic acid amplification testing for *Chlamydia trachomatis* and *Neisseria gonorrhoeae* was negative. Chest CT revealed nodules in bilateral lower lobes, measuring 6.1×4.6 cm on the right and 2.4×1.7 cm on the left. Hypervascularity and cavitation were noted of some lesions (Figure 4).

There was no hilar adenopathy. Etiologies for fungal (histoplasma, cryptococcus, and pneumocystis), and bacterial infections (tuberculosis versus non-tuberculous mycobacterial) were considered, as well as malignant processes (Kaposi’s sarcoma versus AIDS-related lymphoma). Further testing was obtained (Tables 3-4).

**Table 3:** BAL analysis BAL: bronchoalveolar lavage

Component	Result
Appearance	Hazy fluid
Cell count	65% polymorphonuclears, 7% lymphocytes, 27% mono/macrocytes, and 1% eosinophils

**Table 4:** Most relevant BAL infections BAL: bronchoalveolar lavage

Test	Result
BAL routine bacterial culture	Negative
BAL fungal culture	Negative
BAL Legionella culture	Negative
Aspergillus galactomannan antigen	Negative
Nasopharyngeal swab for coronavirus - PCR	Negative
Acid-fast bacilli smear and culture from sputum	Negative

**Figure 5:**
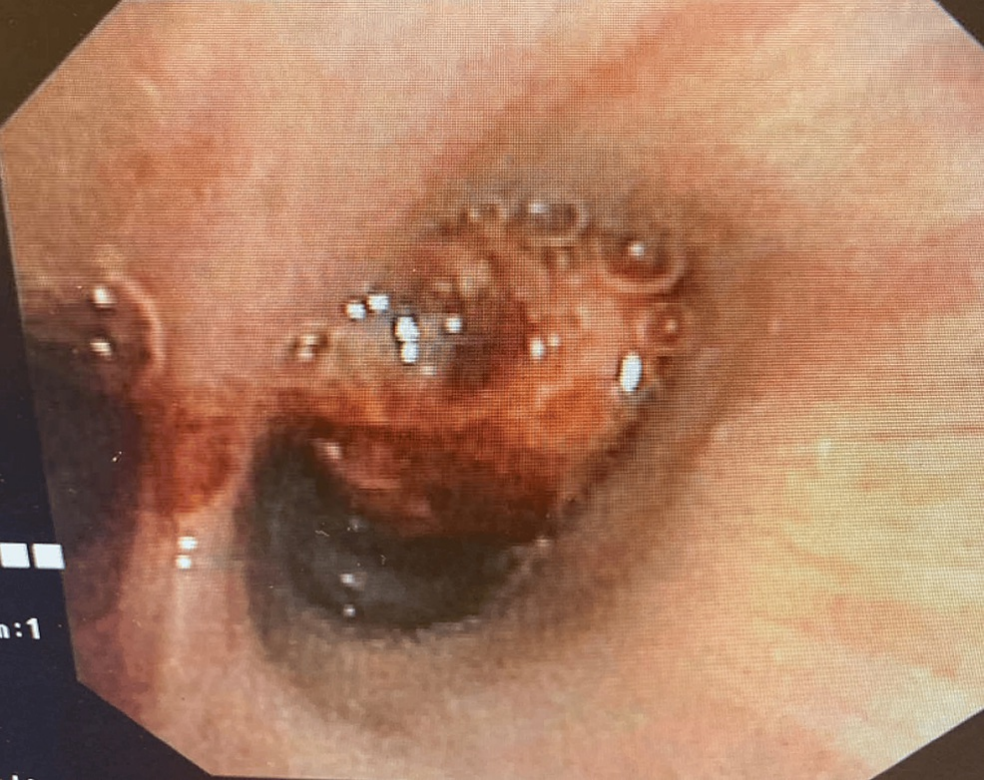
Bronchoscopy revealed a hypervascular mass in the right lower bronchus.

Bronchoscopy revealed a hypervascular lesion in the right lower bronchus suggestive of Kaposi sarcoma, however, biopsy was not obtained at the time of bronchoscopy due to the risk of bleeding (Figure 5).

**Figure 6:**
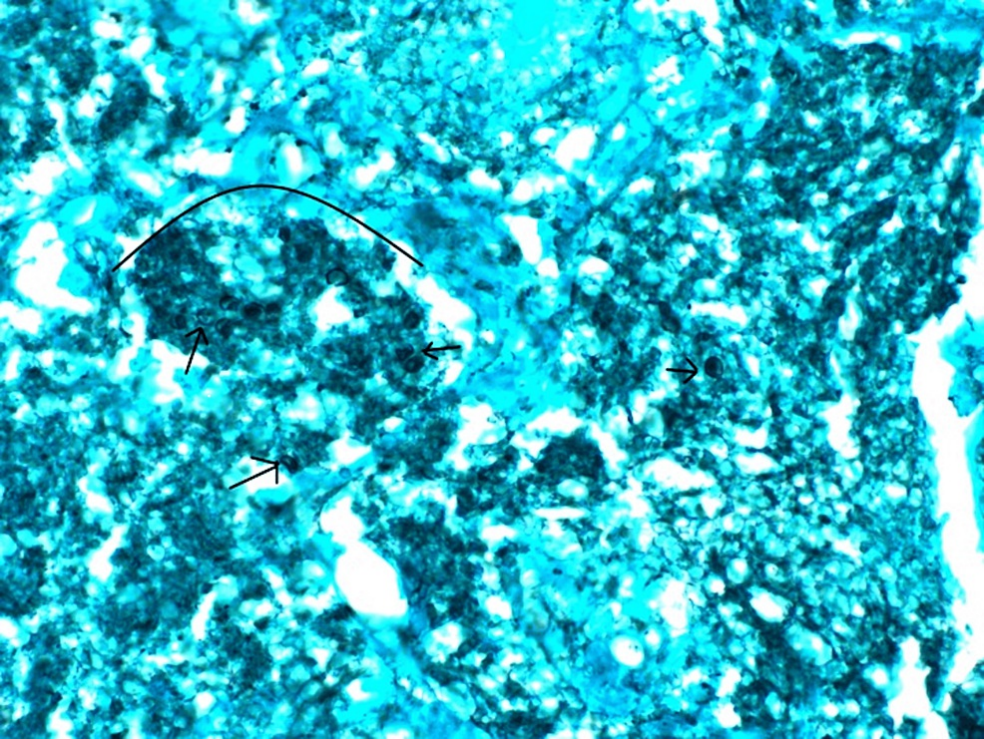
GMS stain with 500x magnification. Grocott methenamine silver (GMS) stain shows the individual organisms (black arrows) and the clustered organisms (black arc). This is referred as “crushed ping-pong ball” forms. Rare central dark dots inside the organisms are morphologically consistent with pneumocystis.

One week later, CT-guided biopsy was performed of a right lower lobe lesion. The biopsy revealed necrotic debris with microcalcifications, intra-alveolar foamy macrophages, and multinucleated giant cells. Grocott methenamine silver (GMS) showed rare yeast forms with a crushed “ping-pong ball” appearance that was compatible with pneumocystis infection (Figure 6).

Pneumocystis pneumonia (PCP) pneumonia was diagnosed. Herpes virus type 8 (HHV-8) stain revealed no HHV-8 in the biopsy specimen. HHV-8 PCR was negative in blood, however due to the lesion seen on bronchoscopy Kaposi sarcoma was not completely ruled out. Initiation of ART is recommended in patients with AIDS-related Kaposi sarcoma.

While the patient's umbilicated lesions on the face and eyelid strongly suggested MC, we considered cutaneous histoplasmosis and cryptococcosis as possibilities due to her new AIDS diagnosis. However, both cryptococcal antigen tests in blood and CSF were negative, and histoplasma in urine was also negative. The biopsy revealed that many of the suprabasal epidermal cells contained ovoid eosinophilic structures, compressing the nuclei (Henderson-Paterson bodies). These structures extended up to a hyperkeratotic stratum corneum, confirming MC. Ophthalmologic evaluation for associated MC lesions of the cornea and conjunctiva were performed with no findings. White patches in the buccal mucosa of the patient were typical for oral candidiasis.

ART with bictegravir, emtricitabine, and tenofovir alafenamide was initiated. MC was expected to eventually remit due to ART; thus, lesions were not removed. Seborrheic dermatitis was treated with selenium sulfide shampoo twice weekly. Oral candidiasis was addressed with fluconazole. PCP was treated with high-dose trimethoprim/sulfamethoxazole (TMP/SMX) for 2 weeks, and daily TMP/SMX was indicated for PCP prophylaxis. No prophylaxis for *Mycobacterium avium* complex infection was recommended since she was immediately started on ART during hospitalization. Due to failure to thrive and weight loss, a dietitian consultation recommended a high-protein diet. Physical and occupational therapies were initiated, and a referral was made for admission to a rehabilitation facility. Subsequently, she continued follow-up in another facility.

## Discussion

MC affecting the eyelids in adults is a well-recognized finding observed in the advanced stages of HIV disease [7,8]. MC was first reported in HIV patients in 1983. This sign was more often noted in the 80s and early 90s when HIV diagnosis was frequently made during later stages of the infection and when available ART was less effective. The prevalence of MC ranges between 10% and 20% in patients with advanced HIV [9]. MC is a skin infection caused by a DNA virus of the Poxviridae family, with two major genotypes identified as MCV1 and MCV2.

There is evidence indicating a higher incidence of MCV2 among HIV patients [10]. Transmission occurs through direct contact with the skin, primarily infecting keratinocytes and typically remaining localized in the epidermis, although conjunctival and corneal involvements have also been reported [11].

MC lesions present as small, domed-shaped, pearly, or pink papules with central umbilication, often asymptomatic, but occasionally pruritic. They may exhibit inflammatory, conglomerated, pedunculated, or erythematous features [9]. In sexually transmitted cases, MC is predominantly found in the abdomen, thigh, and anogenital area, while immunocompromised adult patients may present with atypical locations in the upper body and face, or in an extensive pattern with an increased number and size of skin lesions [10,12]. Of note, MC is rarely on the palms or soles.

While MC is typically self-limited, resolving within 3-12 months in healthy individuals, it can persist for extended periods in patients with HIV infection. Differentials include fungal infections such as disseminated cryptococcosis, which has been reported to coexist with MC [13]. Talaromycosis is also considered, as both can present as umbilicated cutaneous lesions. A biopsy is particularly crucial in HIV patients with extensive umbilicated cutaneous lesions [14]. Eyelid MC in adults usually manifests in advanced HIV cases with a CD4+ cell count approximately below 85 cells/µL [12,15].

Despite the availability of numerous treatments proposed for MC, the lack of a universally accepted treatment due to limited strong evidence necessitates physicians to determine the most suitable approach individually for each patient. Present therapeutic options can be classified as mechanical, chemical, immunomodulatory, and antiviral [16]. The most commonly used therapies are mechanical and chemical therapy, which includes cryotherapy, curettage, cantharidin, and podophyllotoxin.

Some cases have reported that topical imiquimod has favorable outcomes in immunocompromised individuals with extensive, giant, and/or resistant cases of MC [17,18].

Additionally, several studies have reported successful recovery treating MC lesions among HIV/AIDS patients using the antiviral agent cidofovir, administered both intravenously or topically [19,20].

The current recommended approach for treating MC in the setting of HIV patients is with ART. It is believed that restoration of the immune system can lead to improvement of MC lesions in number and size. However, it is important to note that some cases have reported that the initiation of ART can also potentially trigger the onset of MC as a result of the immune reconstitution inflammatory syndrome (IRIS) [21].

As demonstrated by our patient, a diagnosis of HIV requires awareness by the clinician. While the majority of patients with HIV infection have certain “classic” risk factors - men who have sex with men, intravenous drug abusers, those who have sex for money or drugs - it is important to note that other risk groups include those who have sex with more than one partner since the last HIV testing was performed, presence of other sexually transmissible infections (STIs), and having sex with someone without knowing their sexual history; all are indications to perform HIV testing. In our patient, her risk factor was having unprotected intercourse during her long-term relationships with no HIV test performed in between. A Kaiser foundation study published in 2019 revealed that only 43% of non-elderly adults in the US have ever had an HIV test [22].

Our case highlights the importance of incorporating routine HIV testing into a clinician’s daily practice. Another important aspect of our case is the reminder that uncommon presentations of common diseases should trigger an HIV infection evaluation. A few examples would include a diagnosis of zoster in a younger person, extensive seborrhea, and MC of the eyelid in adults [23,24].

## Conclusions

Physicians should be aware that the presence of MC on the eyelids in adults should trigger immediate suspicion of immunosuppression, prompting the ordering of an HIV test. While MC typically resolves within a short period in healthy individuals, it can persist for extended periods in those with HIV infection. Healthcare providers should integrate regular HIV testing and not only recommend this test in individuals with the “classical risk factors”.
